# Experimental Investigation of Hybrid Carbon Nanotubes and Graphite Nanoplatelets on Rheology, Shrinkage, Mechanical, and Microstructure of SCCM

**DOI:** 10.3390/ma13010230

**Published:** 2020-01-04

**Authors:** Furqan Farooq, Arslan Akbar, Rao Arsalan Khushnood, Waqas Latif Baloch Muhammad, Sardar Kashif Ur Rehman, Muhammad Faisal Javed

**Affiliations:** 1 School of Civil and Environmental Engineering, National University of Sciences and Technology (NUST), Sector H-12, Islamabad 46000, Pakistan; aakbar4-c@my.cityu.edu.hk (A.A.); wakas.baloch@gmail.com (W.L.B.M.); kashif@engineersdaily.com (S.K.U.R.); arbabf1@gmail.com (M.F.J.); 2 Department of Architecture and Civil Engineering, City University of Hong Kong, Kowloon, Hong Kong 999077, China

**Keywords:** dispersion, shrinkage, superplasticizer demand, rheological properties, microstructure

## Abstract

Carbon nanotubes (CNTs) and graphite nanoplatelets (GNPs) belong to the family of graphite nanomaterials (GNMs) and are promising candidates for enhancing properties of cementitious matrix. However, the problem lies with their improper dispersion. In this paper graphite nanoplatelets are used with carbon nanotubes for dispersion facilitation of CNTs in cement mortar. The intended role is to use the GNPs particles for dispersion of CNTs and to investigate the synergistic effect of resulting nano-intruded mortar. Mechanical properties such as flexure and compressive strength have been studied along with volumetric stability, rheology, and workability. Varying dosages of CNTs to GNPs have been formulated and were analyzed. The hybrid use of CNTs-GNPs shows promise. Scanning electron microscopy reveals that hybrid CNTs/GNPs are well-suited for use in cement mortar composite performing a dual function.

## 1. Introduction

Cementitious materials are advancing every day. Civil engineering structures are primarily based on cement as the core binder. However, the carbon footprint attached to the production and application forces the researchers to improve its eco-friendliness. With new primary and secondary binders (SCMs) available, there is room for research and development. The concrete matrix can withstand a variety of loads during its serviceable life and shows durability in ordinary environments. However, in aggressive environments, the behavior of cementitious matrices is passive. In the last few decades, most of the research has focused on secondary cementitious materials (SCMs), such as fly-ash (FA), silica fume (SF), blast furnace slag (GGBS), to improve mechanical and durability characteristics. These materials can improve the aforementioned but the resulting incumbent matrices are still passive. To induce smart characteristics and functional properties, such as self-sensing and self-healing, carbon-based nanomaterials are gaining attention developing countries. Carbon nanomaterials or nano-inerts such as carbon nanotubes (CNTs) [1,2], carbon nano-fibers (CNFs) [3], graphite nano-platelets (GNPs) [4], etc., have revolutionized the cement industry. This is due to the high aspect ratio, large surface area and finer nature of these particles that improve the inner packing resulting in a denser cement composite. These modern cements deliver the utmost opportunities to enhance and improve the mechanical aspect of the cement composite. Research has been carried out on these CNTs and GNPs nanomaterials on mechanical properties of cement composite [5,6,7,8]. The success of these materials is owed to their small dosage delivering utmost aspects at the molecular level, causing refinement of pore size, and ultimately enhancing the mechanical properties [9,10,11,12]. 

Research has been carried out in the past for enhancing the mechanical properties of nanocomposite by using a small dosage. Sedaghatdoost et al. used MWCNTs in a small dosage at a ratio of 0% to 0.15% in cement mortar by weight of the binder. It was found that using a small dosage enhances the mechanical properties at elevated temperatures [13]. Moreover, the study found that by using a small concentration of nano-media, (0.1%) significant enhancement in mechanical properties can be achieved. Results showed an 8% enhancement in tensile, 12% in flexural, and 35% in compressive strength, respectively. Larger surface area of nano-materials gives them an edge over the conventional particles such as FA, SF and this gets reflected in improved mechanical strength [14,15,16]. The intrusion of these nano-materials in cement composites regulates the initial hydration, and provides nucleation sites for the hydrates to grow and subsequently improve the mechanical properties [17,18,19]. Surface treatment can be done to further hone the functional properties of nano-inerts in cement. Li et al. [20] used surface-treated multi-walled carbon nanotubes with sulfuric acid (H_2_SO_4_) and nitric acid (HNO_3_) in cement mortar. The author concluded that using surface-treated carbon nanotubes enhanced the overall response in terms of mechanical properties like flexural and compressive strength. Authors re-iterated the fact that using the nanotubes enhances the microstructure and results in a densely packed matrix. Gillani et al. [21] used multi-walled carbon nanotubes by varying concentration in cement composite for improving the mechanical properties and reported that a small concentration of MWCNTs (0.05% by wt. of binder) increases the compressive, flexural and splitting tensile strength by 15.60%, 26.29%, and 20.58% respectively. The mechanical strength of cement-based materials can be improved by using a small quantity of nano-media. However, in order to achieve these increments, the nano-inerts must be adequately dispersed in the host matrix. These nanomaterials have strong surficial forces between them, known as the van der Waals [22]. The presence of these forces leads to the entanglement of these nanoparticles. Further, these nanomaterials are hydrophobic in nature and do not get easily dispersed in an aqueous medium. These strong intraparticle forces make the dispersion difficult but the lack of reactiveness with the host matrix ensures the chemical integrity [23,24]. Improper dispersion, however, creates pockets of entangled and ineffective nano-media which compromises the mechanical strength. 

The dispersion of nano-media in cement improves the mechanical properties but ensures the adequate induction of functional properties, which is the primal goal in modern cement composites. Some studies have indicated towards the use of SCMs to help and facilitate the dispersion of nanotubes. Chaipanich et al. used CNTs with a different concentration in cement fly-ash to make cement fly-ash nanocomposite [25]. The author concluded that using 1% CNTs with 20% fly-ash in cement mortar enhances the compressive strength to about 51.8 MPa. The strength enhancement was a result of improved CNTs dispersion in the host matrix. Moreover, fly-ash cement nanocomposite refines the pore size and dense the distribution with the adjacent matrix. Morsy et al. used nano metakaolin (NMK) as blended materials with a 6% replacement in Portland cement with different concentrations of multi-wall carbon nanotubes (MWCNTs). The author concluded that using (NMK) as replacement increases the compressive strength of mortar by 18% sample and using 6% NMK with 0.02% CNTs by weight increases the compressive strength by 26%, as compared to control [26]. The nano-media was adequately dispersed, resulting in improved mechanical strength. Kim et al. [27] used silica fume as SCM in conjuncture with carbon nanotubes CNTs in cement composite for the facilitation of dispersion for CNTs. The author concluded that using silica fume helps in dispersion for CNTs and leads to a denser packing. The author acknowledged the use of SCM as a dispersion facilitator which caused an increase in compressive strength. However, it was concluded that using silica fume in high dosage can lead to a decrease in compressive strength. Zhou et al. investigated the dispersion effect of CNTs in graphene-based solution (GO) without using surfactant in cement paste. The author concluded adamant enhancement in the mechanical properties of the hybrid mix. This is due to well-dispersed CNTs in GO solution, which ultimately increase mechanical response in terms of compression and flexural by 23.9% and 16.7%, respectively [1].

Dispersing the carbon nano-inerts using carbon-based materials has also been looked into as a viable alternative to SCMs. The use of binary-carbonaceous inerts in cement is deemed useful. The dispersant helps disperse the primary nano-media but it improves the mechanical strength as well. Lu et al. [28] used graphene oxide (GO) for the dispersion of carbon nanotubes CNTs in cement paste and concluded that using CNTs in GO solution facilitates the dispersion of CNTs. Moreover, using GO/CNTs lead to an increase in flexural and compressive strength by 24.21% and 21.13%. The reported improvement is more than using CNTs and GO individually in cement paste [29,30]. Chen et al. [31] used hybrid carbon nanotubes (CNTs) with graphene nanoplatelets (GNPs) in the ratio of (1:1) in cement mortar. The author concluded that using hybrid CNTs/GNPs improves the overall response in mechanical properties as compared to the individual use of CNTs and GNPs. 

A study has thus been developed to identify the effect of varying nano-media (dispersant) on the dispersion of primary nano-media. In the first phase, carbon nanotubes (primary) have been studied in conjuncture with the varying amounts of graphite nano-platelets (dispersant), then vice-versa has been established. The rheological and mechanical properties of hybrid CNTs/GNPs in a self-compacting cementitious system (SCCM) have been studied. Further, the behavior of several matrices is evidenced through microstructural study.

## 2. Experimental Program

### 2.1. Material and Methods

**Figure 1:**
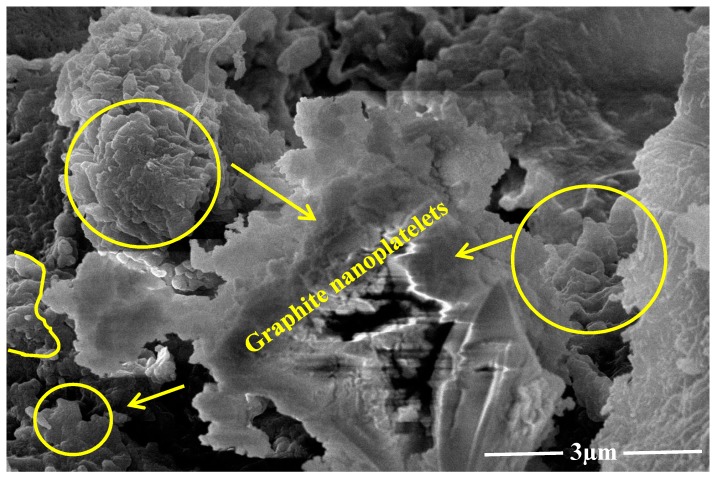
SEM image of exfoliated graphite.

Ordinary Portland cement (OPC) Type 1 Grade 42.5-N and natural sand conforming to ASTM C150 and ASTM C128 were used for casting of specimens [32]. X-ray fluorescence (XRF, JEOL, Tokyo, Japan) was done in order to obtain the elemental composition of all ingredients. The chemical and physical properties of cement and sand are listed in Table 1 and Table 2. Carbon nanotubes were obtained from US Research Nanomaterials, Inc. Graphite nanoplatelets were procured from Daejung Company (GYEONGGI-DO, Korea) and were produced by exfoliation of natural graphite powder, as shown in Figure 1. Properties of nanomaterials are listed in Table 3 and Table 4. Third generation poly-carboxylate ester (PCE) super-plasticizer conforming to ASTM C494 Type F and EN 934-2 [33,34] was used for self-compacting cement mortar properties (SCCM). Acacia gum was also used as a surfactant against the surficial forces to assist the effective dispersion of nano-media. The properties of superplasticizer and acacia gum are listed in Table 5 and Table 6.

### 2.2. Dispersion of Nanomaterials

The dosage of carbon nanotubes with varying concentrations of GNP and vice versa under optimum dosage was incorporated in all the formulations to check the synergistic effect of the hybrid nanocomposite. Shah et al. [35] concluded, that 0.08 wt% MWCNTs as an optimum value by weight of cement. Beyond this optimum value, re-agglomeration occurs in host cementitious matrix. Efforts have been made to improve the dispersion of nanotubes beyond the critical limit of 0.08%, however, re-agglomeration was reported in studies with an adverse effect on mechanical properties [20,36,37].

**Figure 2:**
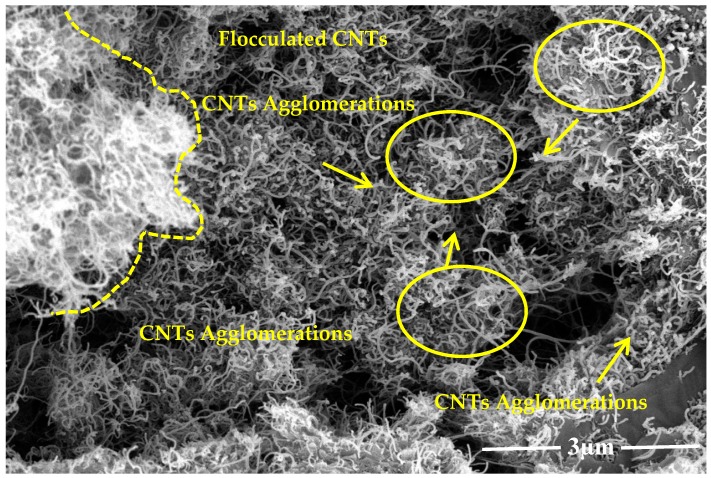
Carbon nanotubes (CNTs) agglomeration without surfactant.

**Figure 3:**
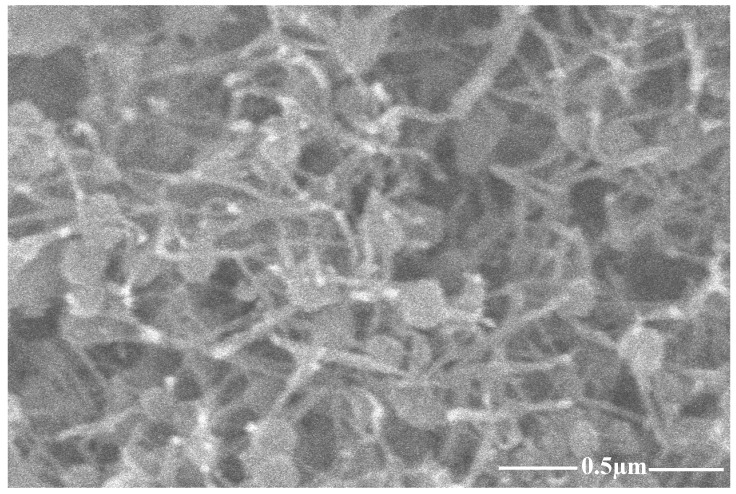
CNTs effect on surfactant.

**Figure 4:**
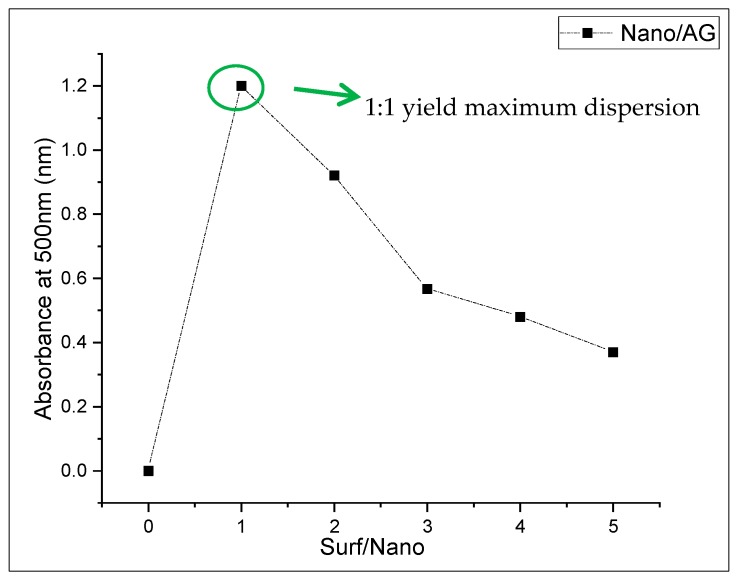
Absorbance of nanocomposite using AG as a surfactant.

Carbon nanotubes and graphite nano-platelets belong to the family of graphite nanomaterials (GNM’s). These nanomaterials are hydrophobic in nature thus difficult to disperse in an aqueous medium. This is due to the presence of strong surficial forces called van der Waals forces. Such agglomerated nanotubes are shown in Figure 2. To mitigate the effect of strong van der Waal surface forces, the use of surfactant with sonication is required [12]. In order to attain effective dispersion that enhances the mechanical properties of the host matrix, it is necessary to use a surfactant. Although many chemicals are used as surfactants, they might have adverse effects on the host matrix. Some natural surfactants have been studied, which help disperse the nano-media in water but also help stabilize the dispersion. One such natural surfactant is acacia gum. Acacia gum used as a surfactant weakens the strong attractive forces through adsorption among graphite nanomaterials. However, it does not affect the freshly hardened properties of the incumbent matrix [12]. Scanning electron microscopy reveals that using a surfactant in nanomedia facilitates the dispersion effect and minimizes the entanglement effect between nanosize media, as shown in Figure 3. This ultimately enhances the mechanical properties of the host matrix [12,37]. In the present study, surfactant to nanomaterials ratios initially chosen were from 1:1 to 1:5. In the first phase, graphite nanoplatelets were used for dispersion facilitation of CNTs, as shown in Figure 1. The surfactant ultrasonication method was used to attain dispersed aqueous solutions. The surfactant to nano-media ratio of 1:1 yielded maximum dispersion, as shown in Figure 4. Aqueous solutions were diluted to the extent which can be used for the preparation of mortar (water demand). A representative sample from the diluted aqueous solution was collected to ascertain the dispersion. The effectiveness of dispersion can also be monitored by using ultraviolet-visible spectroscopy. The absorbance of aqueous solution at 500 nm is generally examined, which remains unaffected under ambient conditions [38]. In the present study, the ratio 1:1 showed maximum absorbance at the set 500 nm scale, thus was selected for the rest of formulations as well. 

### 2.3. Mixing and Preparation of Samples

The mixing of all formulations was carried out accordingly to EN/196-1 standard and is listed in Table 7 [39]. The mixing of all the specimens was done by using a Hobert mixer of 5L capacity at room temperature (25 °C). The mixing regime of formulation was done in three steps: (1) Dry constituent sand and cement were mixed for 30 s, (2) dispersed nanomedia was then added with superplasticizer and water about 80% in Hobert mixer which was then mixed at 145 rpm slow mix for 60 s, (3) superplasticizer and remaining water were added and mixed at 285 rpm fast mix for 150 s. In total 240 s time was used for achieving the SCCM properties. Four formulations were selected, and the concentration of CNTs/GNPs varied from optimum concentration which was also the upper limit (0.08%) to 0% as the lower bound. Before mixing, the dispersion was carried out by using a bath sonicator at 40 kHz for 45 minute. To prevent overheating of suspension, an interval pause of about 30 s was applied. The dispersed aqueous solution with surfactant to nanomaterial (1:1) was diluted to required water demand, then, it was added to cement and sand to develop mortars. The cement to sand ratio and water to cement ratio used in the formulation was kept as 1:1.5 and 0.38.

### 2.4. Slump Test of SCCM

**Figure 5:**
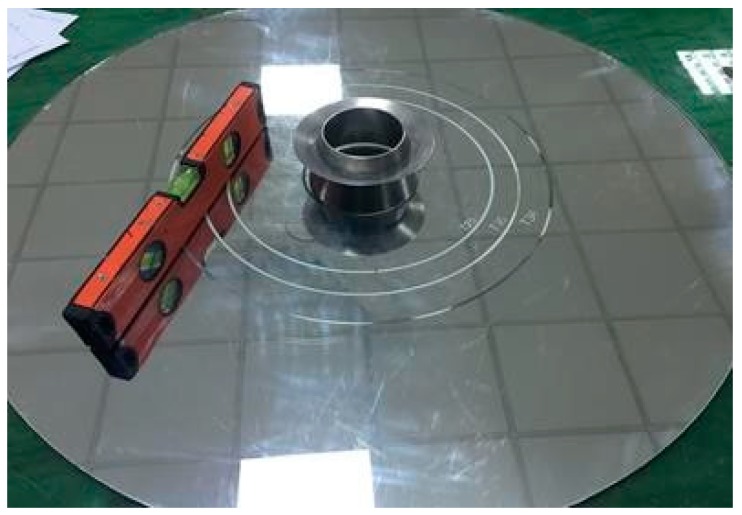
Hagerman mini slump cone apparatus.

For the evaluation of the effect of nanomaterials in self-compacting cement mortar (SCCM), the slump test was carried out using a mini cone apparatus according to EFNARC standard [40], as shown in Figure 5. The incorporation of nanomaterials in a mini cone test has two purposes. Firstly, the addition of nanomaterials with different dosages gives the idea of flowability, and secondly, superplasticizer demand of intruded nanomaterials was calculated. After mixing the formulation, according to the EN/196-1 standard [39], the nanocomposite material was poured into a mini cone slump apparatus having a volumetric dimension of 6 × 7 × 10 cm^3^. The target flow for achieving the flow spread was 30 ± 1 cm was kept constant for achieving SCC/SCCM properties. The flow for SCCM was monitored by varying the dosage of superplasticizer. T_25_ cm spread time was also measured from mini cone slump spread, which was based on Abram’s cone analogy because of the same spread diameter to flow diameter ratios (2.5) of respective cones. Therefore, the flow/spread obtained from a mini cone at T_25_ cm time will be the same as those obtained from T_50_ cm time. Similarly, T_30_ cm flow at T_30_ time for achieving SCCM was also monitored [41,42,43].

### 2.5. Water Absorption and Air Content

Water absorption of graphite nanomaterials cement composite was measured according to ASTM C642 [44]. The mortar specimens after casting were de-molded after 24 and weighted, using the electronic balance of high accuracy and cured with water until testing. After 28 d, the cured specimen was taken out and weighed again in saturated condition by removing surface water using tissue paper or towel. The water absorption of specimens can be found by using the given equation and is expressed in percentage. Luftgheltsprufer testing apparatus was used to determine the air content of all formulation containing nanomaterials according to ASTM C231 [45].

$$water\;absorption\left(\%\right)=\frac{\left(W_{2}-W_{1}\right)}{W_{1}}\;\;\;\;\;\;\;\;\;\;\;\;\;\;\;\;\;\;\;\;\;\;$$

### 2.6. Rheological Properties

The rheological properties of nanomaterials were measured by using Brookfield DV-111 ultra-programmable rheometer. The effect of intruded nanomaterial on the rheological properties was calculated at different shear rates which were operated for 20 s each. Rheological properties of all formulations containing CNTs/GNPs were evaluated by using the Bingham model. The mathematical model is as follows.

$$\tau ={\;\tau }_{{}^{\circ}}+\mu_{\rho }\gamma$$

where τ represents shear stress and $\tau_{{}^{\circ}}$, $\mu_{\rho }$, γ represents yield stress, plastic or apparent viscosity and shear rate [46]. 

### 2.7. Shrinkage Control Investigation

**Figure 6:**
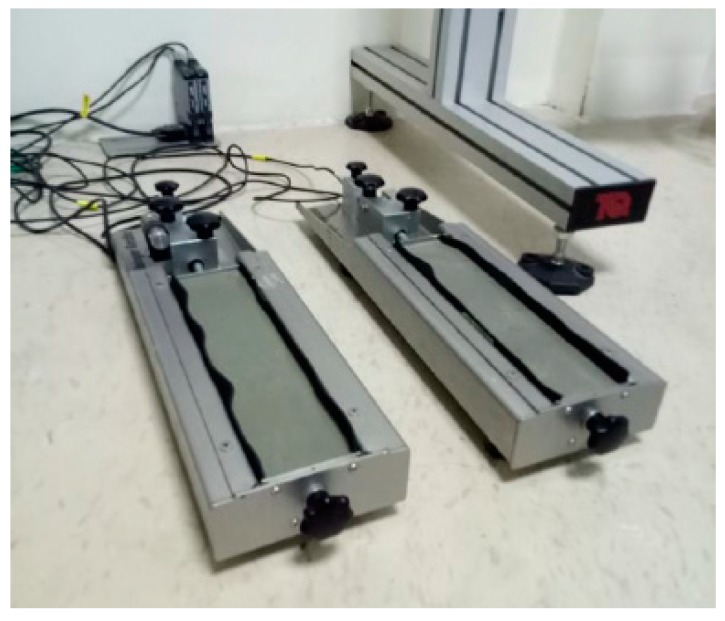
Shrinkage apparatus.

Shrinkage investigation of plain reference and modified mortar with additives was measured through the German modified Schwindrine shrinkage apparatus, as shown in Figure 6. Before the shrinkage measurement, specimens with different concentrations were made through the mixer and then placed in the shrinkage apparatus for 48 hours, in which the samples were uncovered for 48 hours at room temperature with 90% relative humidity.

### 2.8. Mechanical Properties

**Figure 7:**
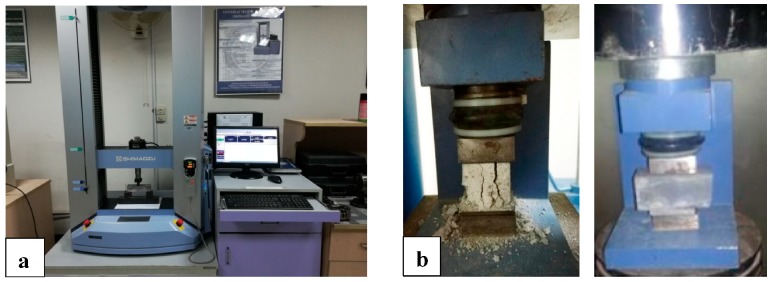
(**a**) Flexural response of nanocomposite, (**b**) compression response of nanocomposite.

The flexural test (Shimadzu, Kyoto, Japan) of nano intruded cement mortar was conducted according to the ASTM C348-14 standard using a bending test [47]. The test of nanocomposite cement mortar was conducted at different ages on 48 specimens in which the loading rate was kept as 0.025 MPa/min shown in Figure 7a. The compressive test was conducted on the broken sample at different ages on 96 specimens conforming to the ASTM C349-14 standard [48] after the flexural test was conducted, as shown in Figure 7b. The test conducted on a load control machine in which the loading rate was kept as 0.25 MPA/min, as shown in Figure 7. 

## 3. Result and Discussion

### 3.1. Dispersion of Nano Intruded Cement Mortar

**Figure 8:**
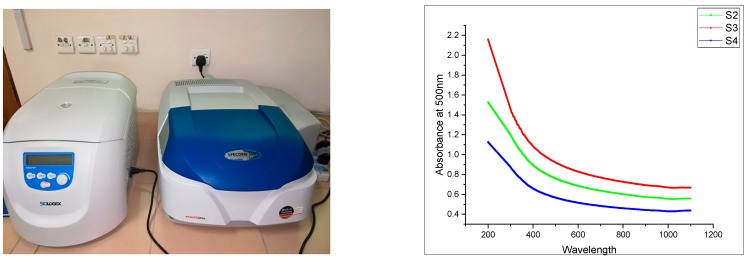
UV-spectroscopy of dispersed nanomaterials.

The dispersion of nano-media plays a vital role in enhancing the mechanical properties of SCCM. The dispersion mechanism of nanomaterials with a surfactant to nanomaterials ratio of (1:1) yield maximum dispersion, as shown in Figure 8. It can be seen that the specimen S3 containing carbon nanotubes and graphite nanoplatelets (concentration of CNTs and GNPs is 4% and surfactant is 1 %.) gives maximum absorbance at 500 nm. This is due to the synergistic effect of the nanocomposite. A similar trend was observed in a study conducted by Lu et al. [28]. They dispersed CNTs in graphene oxide solution and obtained maximum absorbance value in UV-vis spectroscopy (Analytik Jena, Jena, Germany) at a similar proportion of nano-materials [28]. 

### 3.2. Superplasticizer Demand and Slump Flow Test

**Figure 9:**
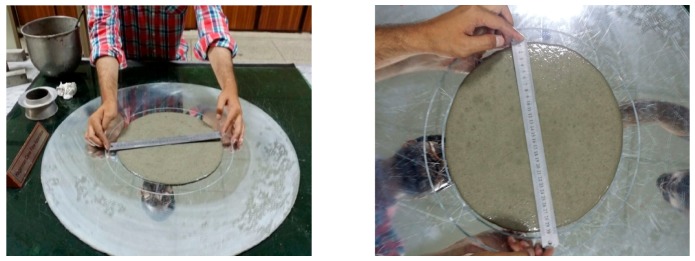
Flow test of self-compacting cementitious mortar.

**Figure 10:**
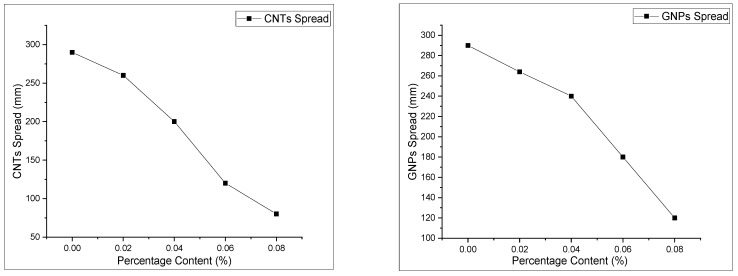
CNTs and GNPs spread using a mini-slump cone by varying different dosages.

The effect of nanomaterials on workability and flowability was adjusted by using a trial method and varying super-plasticizer demand. The system for measuring the flow is shown in Figure 9. The variable content of nano-composites had an effect on the flowability of the host matrix. In the first phase of the test, the optimum dosage of carbon nanotubes with cement paste was selected, and the concentration of GNPs was varied for making hybrid nano-composites. The workability and fluidity of these nanomaterials were then determined by the procedure, as shown in Figure 10. It can be seen that when using nanomaterials, the fluidity as well as the workability of cement mortar decrease. The formulation with a maximum dosage of 0.08% by wt of binder (only concentration of carbon nanotubes (CNTs) and graphite nanoplatelets (GNPs) in conventional mortar) shows an adamant decrease in slump values of about 72% and 32%, respectively, as shown in Figure 10. The possible reason for the reduction in slump value can be associated with strong van der Waals forces and agglomeration of CNTs. Moreover, the concentration in terms of specific gravity of CNTs is more as compared with GNP, thus a large amount of SP is required to overcome the intramolecular force.

**Figure 11:**
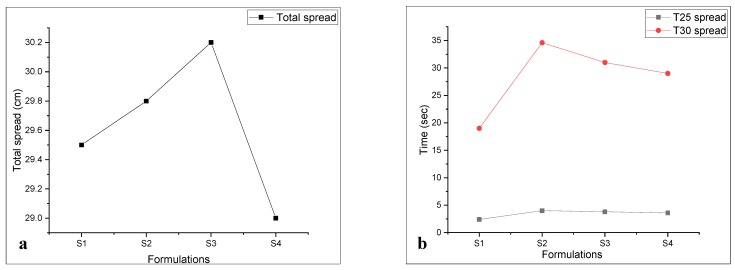
(**a**) T_25_ and T_30_ spread of hybrid nanocomposite, (**b**) total spread of hybrid nanocomposite.

T_25_ and T_30_ cm time and total spread 30 ± 1 of the hybrid nanocomposite were also recorded, as shown in Figure 11a. T_25_ spread defines the viscosity and T_30_ defines the yield stress of respective formulations [49,50,51]. The poly carboxylic ether (PCE) superplasticizer is the key parameter in describing the evaluated properties of self-compacting mortar (SCCM). The total spread of 30 ± 1 was achieved by varying the dosage of polycarboxylic ether with total time, as shown in Figure 11b. Every mix required some amount of super-plasticizer to get the required flow [51,52]. The high super-plasticizer demand of mixture is also associated with the packing density of matrix and inter-particle resistance that develops as a result of incompatibility of cement, sand, and the super-plasticizer [52]. These nano-media fill up the micro/meso-pore which enhance the packing density but in doing so significantly affect the superplasticizer demand.

**Figure 12:**
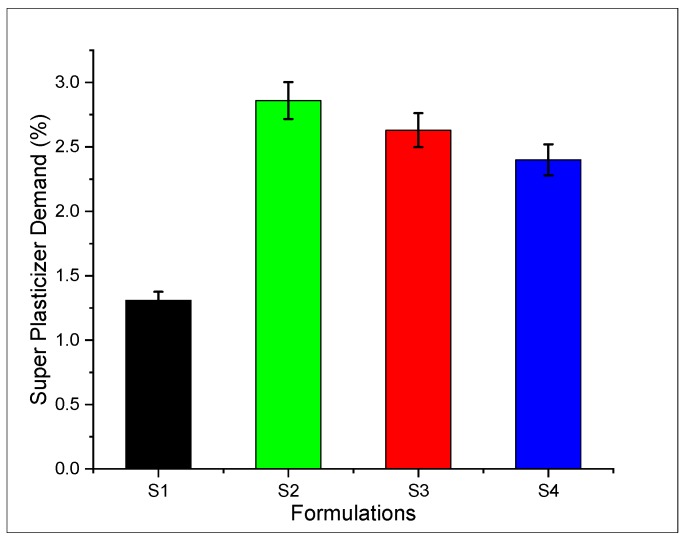
Superplasticizer demand of SCCM.

The addition of nanomaterial improves the packing density of matrix which, in turn, requires a higher amount of plasticizer to achieve the flow. It was found that the superplasticizer demand of specimen S2 (specimen containing only carbon nanotubes with 0.08% concentration) is more than specimen S4 (specimen containing only graphite nano-platelets with 0.08% concentration). Hence, the formulations containing CNTs require a higher content of SP to achieve the required flow as compared to those containing GNPs as shown in Figure 12. This is due to a high aspect ratio (length/diameter) of CNTs compared to GNPs. The particle to particle resistance at a nano-level sometimes increases the demand of SP as well. Similarly, the increasing demand of SP in the case of CNTs can also be attributed to the phenomenon, that to overcome the friction between individual nanotubes, it requires a greater amount of SP. The lubrication effect of CNTs is less than GNPs, thus requiring more superplasticizer demand. The nano-materials adsorb super-plasticizer during mixing in a Hobart mixer, which shows an adverse effect on flowability and results in increasing super-plasticizer demand. For example when the concentration of CNTs is at optimum the adsorption effect dominates, which explains the increasing demand of superplasticizer [52,53]. The higher resistance between individual nanotubes might be associated with a larger aspect ratio, which ultimately leads to hefty super-plasticizer demand, as shown in Figure 12.

### 3.3. Linear Shrinkage Protocols

**Figure 13:**
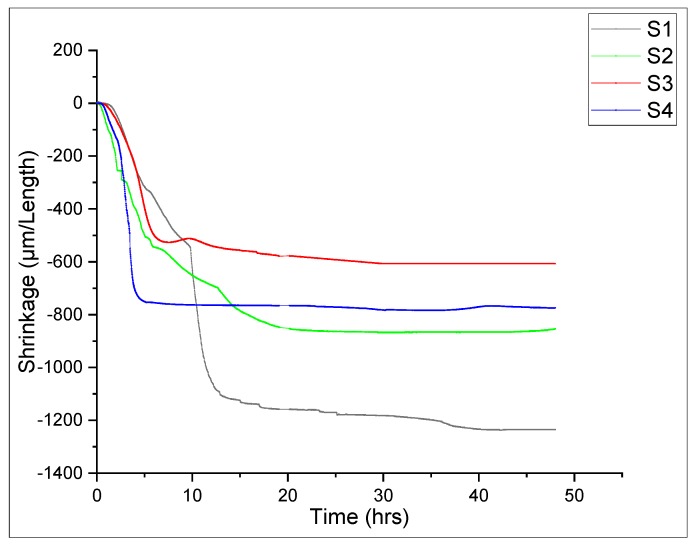
Shrinkage measurement of SCCM.

Shrinkage behavior of reference (S1) and modified mortar (S2, S3, and S4) was measured through the German modified Schwindrine shrinkage apparatus. Modified specimens containing nanomaterials depicts significant reduction in shrinkage response, as shown in Figure 13. The increase in shrinkage response is mainly due to the presence of meso-pores (diameter < 50nm). The increase in the amount of these meso-pores increases the shrinkage response of a particular cement matrix. It can be seen in Figure 11 that the shrinkage of modified specimen decrease as compared to S1. Formulation S3 showed a 51% decrease in shrinkage response as compared to control sample S1. This substantial reduction in shrinkage response of nano-modified samples is due to the refinement of pore size and the filler effect of these nano-materials. The literature suggests that the presence of nano-materials in cement helps the hydration products grow [54]. These nano-materials can act as a nucleation site and provide a platform for hydrates, such as calcium silicate hydrate gel (CSH), to grow. Reduction in shrinkage of modified mortar with an additive is adamant evidence of refinement of pores within the microstructure and presence of high-density C-S-H gel. Moreover, shrinkage deals with water content and has little influence on mechanical properties [55].

### 3.4. Air Content and Water Absorption of Nanomaterials

**Figure 14:**
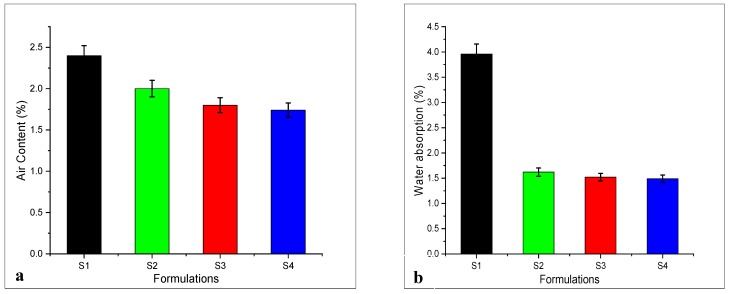
(**a**) Air content of SCCM, (**b**) water absorption of SCCM.

Air content and water absorption of the cement matrix are associated with the pore size. In this study, plain mortar and modified mortar with additives were used which show a significant reduction in pores by nano intrusion, as shown in Figure 14. Air content, as well as water absorption of modified mortar S4 (GNPs:8%), were significantly reduced by 25% and 62% from the control, sample which is due to nano-size materials and surface size of graphite nanoplatelets. The intrusion of these nanomedia significantly reduces the overall response in terms of air content and water absorption. Specimens S2 and S4 also show a decrease in air content and water absorption which is 13% and 23% when compared with control S1 sample, see Figure 14a. GNPs also show reduction in water absorption as compared to reference specimen. Specimens S2 and S3 show a 57% and 59% reduction in overall water absorption, see Figure 14b. These nanomedia provide a nucleation site and make dense packing within the matrix, they also act as a filler material. During the mixing process, these nanomedia fill the pore size within the matrix, and they act as reinforcing agents. Almost all the specimens show a remarkable reduction which is an adamant proof that these nano-sized particles fill the voids and enhance the microstructure with a dense gel.

### 3.5. Rheological Properties

**Figure 15:**
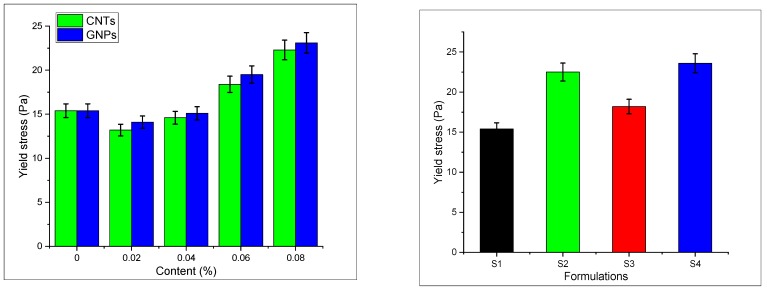
Yield stress of SCCM.

**Figure 16:**
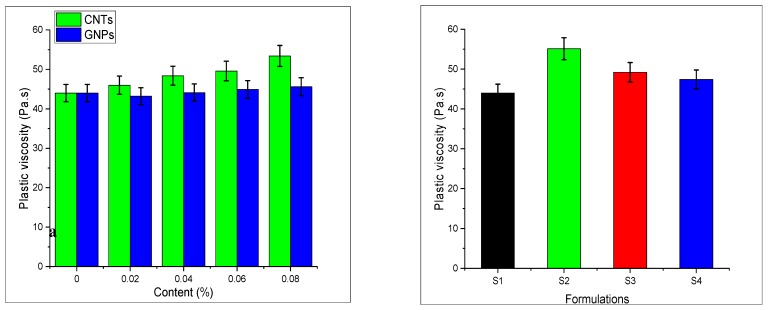
Viscosity of SCCM.

Rheological properties of hybrid CNTs/GNPs samples were estimated by using a Brookfield apparatus. The flowability and workability of the studied mixes varied upon the addition of nano-media, thus making the study of rheological properties important. The procedure adopted is as per the study performed by Nehdi et al. [46,56]. Rheological properties of plain and modified mortar are shown in Figure 13 and Figure 14. It can be seen that the yield stress and the viscosity of modified mortar increase by changing the concentration up to optimum value by 50% and 27%, respectively. The viscosity and yield stress of CNTs and GNPs are shown in Figure 15 and Figure 16.

Yield stress is the value of critical stress after which the material starts to flow [57]. This is due to the denser packing of the matrix by nano-material. S2 and S4 samples show an increase in yield stress. The possible reason is the formation of agglomerated and entrapped water molecules in the flocculated structure of nanomaterials. Due to the van der Waals forces and electrostatic interactive forces, water molecules get entrapped inside the agglomerates/bundles of nanomaterials and reduce the quantity of free available water. A similar trend was observed in other nanomaterials as well Rehman et al. [58] study using graphene and determine the rheological properties and found that entrapped water molecules significantly alter the rheological properties. This free available water is a dominating factor for determining the yield stress. Formulation S3, however, shows a decremented pattern. This is due to the availability of free water due to good dispersion of hybrid mix [28]. The electrostatic repulsion of hybrid mix agglomeration and flocculation is minimal, therefore free water molecules are available, which reduces the yield stress.

Viscosity is resistance against the flow of Newtonian fluids. The viscosity of analyzed cement mortars is shown in Figure 16. Due to the presence of nanomaterials both CNT and GNP plastic viscosity of mortar mix significantly enhance as compared with the control sample. It can be seen that in the cement composite containing the maximum content of carbon nanotubes (S2), viscosity is more as compared to remaining formulations. The addition of CNTs in the cement matrix accelerates the re-agglomeration due to a high aspect ratio. CNTs are adsorbed on the cement matrix through intermolecular forces, thus increasing the hindrance of matrix and enhance the viscosity. On the other hand, S3 formulation shows a decrease in viscosity value as compared with CNTs mixture. This is due to the denser packing of GNP within the matrix [54].

### 3.6. Flexural Strength

**Figure 17:**
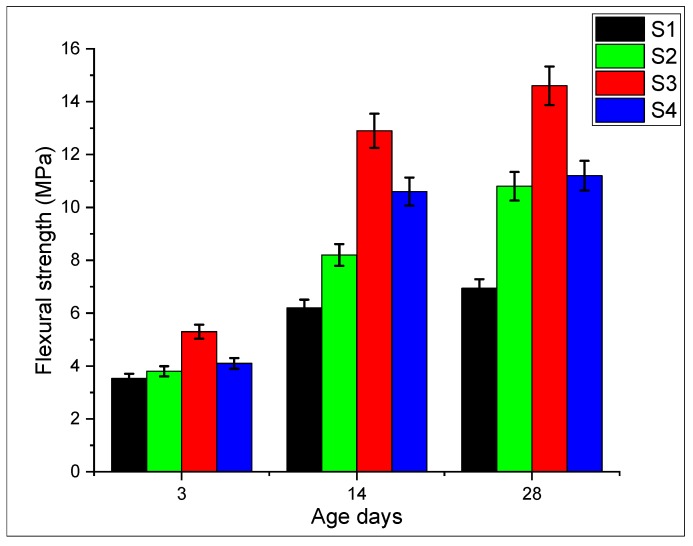
Strength of nanocomposite modified mortar.

**Figure 18:**
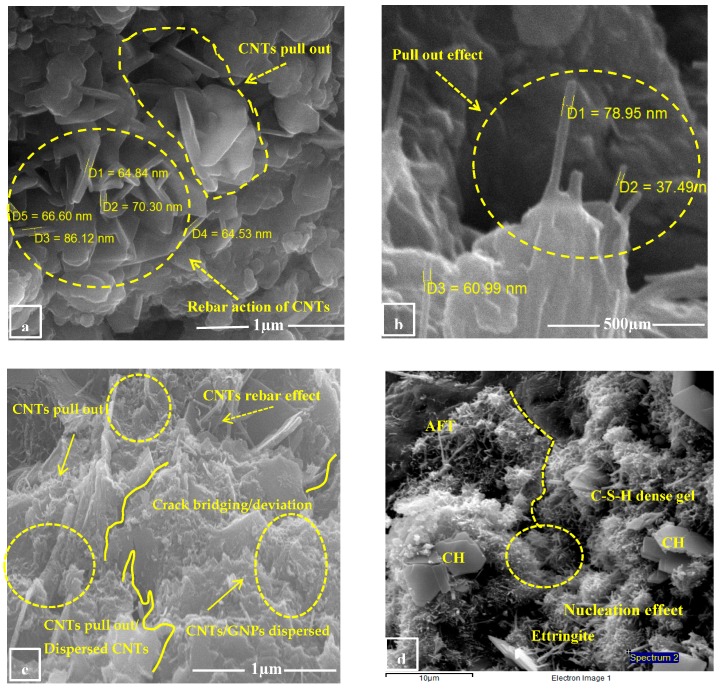
(**a**,**b**) Rebar and pull out effect of CNTs, (**c**) crack deviation and pull out of CNTs, (**d**) crack discontinuity and presence of dense calcium silicate gel with AFT.

The flexural strength of carbon nanotubes in conjunction with graphite nanomaterials on cement mortar is presented in Figure 17. The flexural strength of nano intruded cement mortar increases with respect to the control sample S1. Moreover, formulation S3 shows maximum enhancement in strength by 110% as compared to the S1 control specimen in which the concentration of CNTs and GNPs is equal. Specimen S2 and S4 adamantly show an increase in flexural response which is 56% and 61%, respectively. This is due to the load-carrying capacity between nanomaterials and with the adjacent matrix. Furthermore, GNPs facilitate the dispersion of carbon nanotubes causing an increase in overall strength and can be seen in Figure 8. Further micro-graphical study indicated that these nano-materials preserve the load-bearing area by crack bridging and crack blunting phenomena, see Figure 18c. The nano-tubes bridge across the cracks thus increasing the load-carrying capacity of the incumbent matrix. GNPs effectively blunt (diversion) the crack thereby reducing the accumulation of cracks. The crack bridging capability of CNTs can be witnessed in the micrographs shown in Figure 18. The increase in the tensile strength of hybrid specimen S3 can be attributed to the rebar (reinforcing) action of carbon nanotubes, as shown in Figure 18a,b, which ultimately enhances the mechanical strength. It can be seen in Figure 18c,d that the cracks are deviated and discontinuous. Similar insights can be found in the literature as well. The duality of reinforcing action by nanotubes and graphite nano-platelets is the synergetic effect. Nanotubes bridge the cracks while the graphite nano-platelets blunt them. Pulled out nanotubes can also be seen in the micrographs, see Figure 18b. The pull-out failure occurs if these nanotubes bridge the cracks, but the load increases to a level where the anchorage force is not sufficient to keep the nanotubes effective. The pull-out effect of the nanotube evidences the reinforcing action. This is also observed by various other researchers. Rehman et al. conducted a study on GNP and highlighted that GNPs blocked the growth of cracks at the nanoscale [58]. 

### 3.7. Compressive Strength 

**Figure 19:**
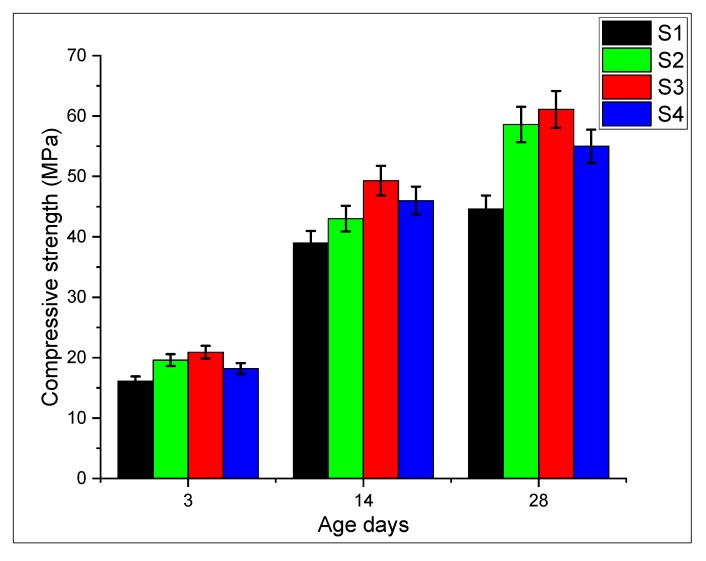
Strength of nanocomposite modified mortar.

**Figure 20:**
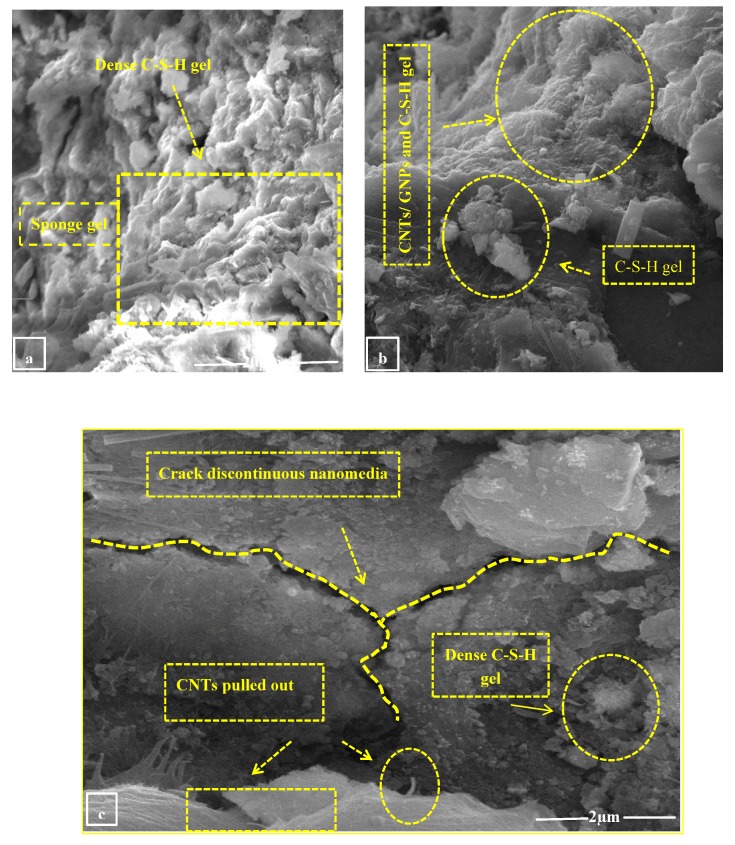
(**a**) and (**b**) Dense sponge C-S-H gel, (**c**) crack discontinuity by nanomedia.

The compressive strength of nano intruded cement mortar at different ages is shown in Figure 19. It can be seen that using carbon nanotubes in conjunction with graphite nanoplatelets increases the overall mechanical response of cement mortar with respect to the control sample. However, S3 shows the maximum enhancement in compressive strength of about 37% as compared to the S1 specimen at 28 days. Similarly, formulations S2 and S4 also show enhancement in compression properties which is 31% and 23%, respectively. This is due to the synergistic effect of well dispersed CNTs/GNPs which enhance the load-carrying capacity of matrix and subsequently the mechanical properties. The intrusion of nano-size material in the modified matrix improves the mechanical response, which is due to denser packing within the matrix and enhanced the load-carrying capacity of the modified matrix. Dense packing of nanomaterial at different scale is shown in figures. It can be seen that calcium silicate gels (C-S-H) exist in the form of a dense sponge with the cement phase, which contributes to strength due to the hydration of cement. These hybrid nanomaterials adhered to dense sponge C-S-H gel, which accelerate the hydration, strengthened the matrix, as shown in Figure 20a,b. Formulations S2 (CNTs with 0.08% by wt of binder) and S3 (hybrid) show maximum compressive strength. The compressive strength of hybrid is, however, maximum as compared with all the other specimens. It is related with the good dispersion of the hybrid mix. Furthermore, the synergetic effect of CNTs and GNPs (crack bridging and blunting) improves the compressive strength. It can be seen in Figure 20 that CNTs pull out from the sample. The pull-out failure indicates that the CNTs were actively reinforcing the matrix (see Figure 18a,b), coping against the cracks. The dispersed CNTs also act as nucleation sites and provide a platform for hydration products to grow, which increases the strength at early and late ages. Furthermore, varying sizes of CNT and GNP provide better packing and filling (filler effect) which enhances the compressive strength [2]. The intrusion of these nanomedia provides crack deviation which can be seen in Figure 20c. These nano media act as filler material and enhancing the load-carrying capacity. This is due to the rebar action and pull-out effect of nanomedia which ultimately take the maximum load and deviate the crack from the original path. as shown in Figure 20c.

## 4. Conclusions

The intrusion of the hybrid CNTs/GNPs in a cement mortar on fresh and hardened properties was investigated and the following conclusions were drawn.

1. The results reveal that using carbon nanotubes with graphite nanoplatelets decreases the workability of nano intruded cement mortar, which ultimately requires more superplasticizer demand for achieving a total spread of self-compacting cementitious mortar within the desired range.
2. Volumetric stability or shrinkage response of formulations containing nano-media improved.
3. Though a varying trend is achieved for viscosity and yield stress. It is fair to say that yield stress and viscosity increase upon the induction of nano-media.
4. Flexural and compressive strength of modified specimen increase in comparison to the modified samples. However, the hybrid formulation performed the best.
5. Scanning electron microscopy reveals the crack bridging phenomena of hybrid CNTs/GNPs with the adjacent matrix.

## Figures and Tables

**Table 1:** Properties of ordinary Portland cement (OPC).

Chemical Composition	Content (%)	Physical Properties	Content (%)
CaO	65.11	Insoluble residue (% by mass)	0.54
SiO_2_	19.17	Specific gravity (g/cm^3^)	3.18
Al_2_O_3_	4.96	Particle size (d_50_) (mm)	16.42
Fe_3_O_4_	3.21	Specific surface area (m^2^/g)	0.86
MgO	2.23	Loss on ignition (% mass)	2.24
MnO + K_2_O	0.55		
TiO_2_	0.28		
P_2_O_5_ + Na_2_O	0.64		

**Table 2:** Properties of sand.

Physical Properties of Sand	Fineness Modulus	Specific Gravity (SSD)	Water Absorption	D_50_ (μm)
	2.25	2.66	1.54%	0.53

**Table 3:** Properties of MWCNTs.

External Diameter (nm)	Internal Diameter (nm)	Length (μm)	Purity (%)	Specific Surface Area (m^2^/g)	Ash Content (wt.%)	Density (g/cm^3^)
20–30	5–10	10–30	> 97	110	< 1.5	2.1

**Table 4:** Properties of graphene nanoplatelets (GNPs).

Specific Surface Area (cm^2^/g)	Particle Size Analysis (μm)	Specific Gravity
154	6.78	1.62

**Table 5:** Properties of superplasticizer.

**Master Glenium ^®^51**	**Aspect**	**Relative Density**	**pH**	**Chloride Ion Content**
	Light Brown Liquid	1.08 ± 0.01 at 25 °C	≥ 6	< 0.2%

**Table 6:** Physical and chemical properties of acacia gum.

Chemical Composition	CaO	SiO_2_	Al_2_O_3_	Fe_2_O_3_	MgO	SrO	K_2_O	Cr_2_O_3_
Content (%)	48.9	–	–	20.3	–	15.22	6.89	6.43
**Physical Property**	**Specific Gravity** **(g/cm^3^)**	**Insoluble Residue** **(% mass)**	**Specific Surface** **area (m^2/^g)**	**Particle** **Size (D_50_) (µm)**	**Loss on Ignition** **(% mass)**			
Content	1.49	5.89	36.97	208	–			

**Table 7:** List of self-compacting cementitious system (SCCM).

S.No.	Formulation	CNTs (%)	GNPs (%)	W/C	SP % Binder
1	S1	0	0	0.38	1.8
3	S2	0.080	0.000	0.38	2.86
4	S3	0.040	0.040	0.38	2.61
5	S4	0.000	0.080	0.38	2.48
